# Herbal Medicines for Treating Metabolic Syndrome: A Systematic Review of Randomized Controlled Trials

**DOI:** 10.1155/2016/5936402

**Published:** 2016-06-19

**Authors:** Soobin Jang, Bo-Hyoung Jang, Youme Ko, Yui Sasaki, Jeong-Su Park, Eui-Hyoung Hwang, Yun-Kyung Song, Yong-Cheol Shin, Seong-Gyu Ko

**Affiliations:** ^1^Department of Korean Preventive Medicine, Graduate School, Kyung Hee University, Seoul 02447, Republic of Korea; ^2^Department of Preventive Medicine, College of Korean Medicine, Kyung Hee University, Seoul 02447, Republic of Korea; ^3^Department of Healthcare Safety Research, National Evidence-Based Healthcare Collaborating Agency, Seoul 04554, Republic of Korea; ^4^Third Division of Clinical Medicine, School of Korean Medicine, Pusan National University, Yangsan 50612, Republic of Korea; ^5^Department of Korean Medicine Rehabilitation, College of Korean Medicine, Gachon University, Incheon 21565, Republic of Korea

## Abstract

*Objective*. The aim of this systematic review is to evaluate the efficacy and safety of herbal medicines in the management of metabolic syndrome.* Materials and Methods*. On December 9, 2015, we searched PubMed, EMBASE, Cochrane Library, SCOPUS, AMED, CNKI, KoreaMed, KMBASE, OASIS, and J-STAGE with no restriction on language or published year. We selected randomized controlled trials that involved patients with metabolic syndrome being treated with herbal medicines as intervention. The main keywords were “Chinese herbal medicines”, “metabolic syndrome”, and “randomized controlled trials”. Herbal substances which were not based on East Asian medical theory, combination therapy with western medicines, and concurrent diseases other than metabolic syndrome were excluded. The risk of bias was assessed by Cochrane's “Risk of Bias” tool. The protocol or review was registered in PROSPERO (an international prospective register of systematic reviews) (CRD42014006842).* Results*. From 1,098 articles, 12 RCTs were included in this review: five trials studied herbal medicines versus a placebo or no treatment, and seven trials studied herbal medicines versus western medicines. Herbal medicines were effective on decreasing waist circumference, blood glucose, blood lipids, and blood pressure.* Conclusion*. This study suggests the possibility that herbal medicines can be complementary and alternative medicines for metabolic syndrome.

## 1. Background

Metabolic syndrome is a complex disorder consisting of central obesity, hyperglycemia, hypertension, and hyperlipidemia [1]. There have been different diagnosis criteria for metabolic syndrome after it was first being defined by the World Health Organization (WHO) in 1998 [2]. The most commonly accepted definition uses the criteria suggested by the American Heart Association/National Heart Lung and Blood Institute (AHA/NHLBI).

According to the AHA/NHLBI, metabolic syndrome is technically diagnosed when three or more of the following five conditions are met: (1) waist circumference (WC) ≥102 cm in men and ≥88 cm in women, (2) triglycerides (TG) ≥150 mg/dL (1.7 mmol/L) or being on drug treatment for elevated triglycerides, (3) high density lipoprotein cholesterol (HDL-C) <40 mg/dL (1.03 mmol/L) in men and <50 mg/dL (1.3 mmol/L) in women or being on drug treatment for reduced HDL-C, (4) blood pressure (BP) ≥130 mmHg systolic blood pressure (SBP) or ≥85 mmHg diastolic blood pressure (DBP) or being on antihypertensive drug treatment, for a patient with a history of hypertension, and (5) fasting plasma glucose (FPG) ≥100 mg/dL or being on drug treatment for elevated glucose [3]. However, the criterion for waist circumference is slightly different for each country and race.

Each metabolic risk factor is associated with one another, and together the risk factors promote atherosclerotic cardiovascular disease [4]. The main underlying risk factors for metabolic syndrome are abdominal obesity and insulin resistance [5, 6]. Therefore, preventing atherosclerotic cardiovascular disease by controlling waist circumference and insulin resistance is the key to managing metabolic syndrome. Clinically, each treatment of hyperglycemia, hypertension, and hyperlipidemia is prescribed according to each patient's state.

However, ACE inhibitor that is a drug used for hypertension, including enalapril and captopril, may cause adverse events such as cough, increased serum creatinine, headache, and skin rash [7, 8]. It has also been shown that metformin, a drug used to treat type 2 diabetes mellitus, can induce gastrointestinal symptoms and lactic acidosis [6]. Therefore, herbal medicines showing evidence of safety and efficacy can be alternative treatments for metabolic diseases.

Although there are several reviews of herbal medicines for obesity [9, 10], hypertension [11], and type 2 diabetes mellitus [12], systematic review for metabolic syndrome has not been conducted yet. This study, however, reviews not only a single disease but also metabolic syndrome as a whole. The aim of this study is to evaluate the efficacy and safety of herbal medicines to help manage metabolic syndrome.

## 2. Materials and Methods

### 2.1. Data Source and Search Strategy

#### 2.1.1. Data Source

This study included the following databases: PubMed, EMBASE, Cochrane Library, SCOPUS, AMED, China National Knowledge Infrastructure (CNKI), KoreaMed, KMBASE, OASIS, Electronic (J-STAGE), and Japan Science and Technology Information Aggregator.

#### 2.1.2. Search Strategy

The study used herbal medicine, metabolic syndrome, and randomized controlled trials for the basic search terms. A search strategy in PubMed is shown in Table 1. Language and publication date were not restricted. The date for the search was December 9, 2015. This review's protocol was registered in PROSPERO (an international prospective register of systematic reviews) (registration number: CRD42014006842).

### 2.2. Inclusion Criteria

#### 2.2.1. Study Design

Randomized controlled trials (RCTs) were included regardless of blinding. Other designs such as* in vivo*,* in vitro*, case report, and retrospective study and thesis were excluded.

#### 2.2.2. Participants

Participants were patients with metabolic syndrome and there was no restriction to sex or age. Diagnosis criteria of metabolic syndrome were restricted to international or national standard criteria such as AHA/NHLBI, National Cholesterol Education Program-Adults Treatment Panel (NCEP-ATP), and International Diabetes Federation (IDF) and should be clearly described in Section 2. Chinese pattern identification was optional. Concurrent diseases with metabolic syndrome were excluded.

#### 2.2.3. Interventions

Single or mixed herbal medicines with East Asian medical theory were included. Traditional Chinese Medicine, Traditional Korean Medicine, and Japanese Kampo Medicine are regarded as East Asian medicine. Ayurvedic medicine, crude plant, food, and dietary supplement were excluded. Combination therapy with western medicines, acupuncture, and moxibustion was also excluded. Exercise, diet-control, and health education were not restricted if they were applied to both intervention and control groups.

#### 2.2.4. Comparisons

There was no special restriction on comparisons. Placebo, no treatment, active-control, exercise, diet-control, health education, and usual care were allowed as control groups. Active-control means western medicines for metabolic syndrome, or herbal medicines other than intervention.

#### 2.2.5. Outcome Measures

The primary outcome measures were WC, FPG, TG, HDL-C, SBP, and DBP which are clinical parameters of metabolic syndrome. Secondary outcome measures included body mass index (BMI), body weight, hemoglobin A1c, 2-hour postprandial glucose (2 hPG), total cholesterol (TC), low density lipoprotein cholesterol (LDL-C), and clinical effective rate. Trials that assessed one or more outcome measures were included. However, trials which measured level of hormone or enzyme, such as leptin and adiponectin, were excluded in this review.

### 2.3. Study Selection and Data Extraction

#### 2.3.1. Selection of Literature Articles

**Figure 1:**
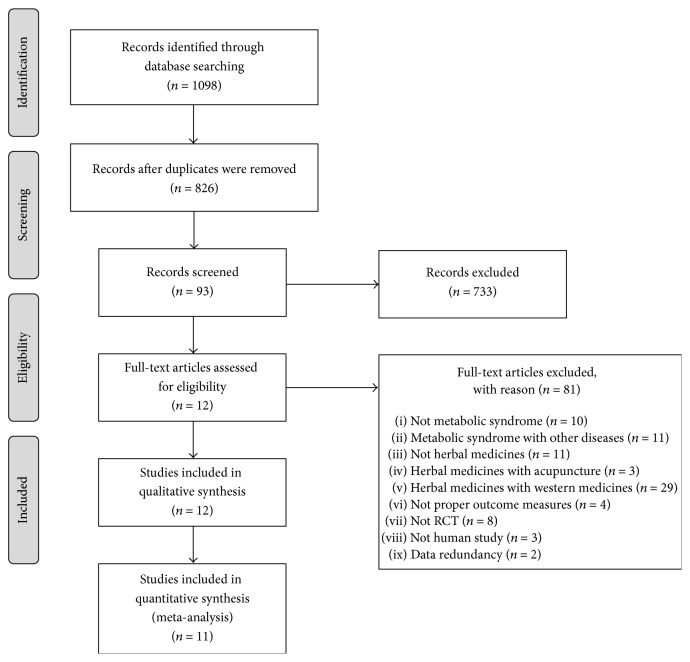
The PRISMA flow diagram of study selection.

After excluding any duplication of literature reviews from 10 databases, two authors (S. Jang and J.-S. Park) reviewed titles and abstracts for the first exclusion. Then, full texts of the selected literature articles were subject to another review before the final selection of literature articles was made to make sure each article qualified using the inclusion criteria for this study. For excluded literature articles, the reason for exclusion was recorded. When two authors showed a difference of opinion, a third author (B.-H. Jang) intervened to help come to an agreement. The entire process was displayed by generating a flow diagram in PRISMA (Preferred Reporting Items for Systematic reviews and Meta-Analyses) (Figure 1).

#### 2.3.2. Data Extraction

One author (S. Jang) conducted data extraction, and a different author (Y. Ko) reviewed the data. Items extracted from each trial include the following: (1) general characteristics of the study: author, published year, language, and country; (2) participants: sample size, sex, and age, Chinese pattern identification; (3) interventions: intervention, compositions of intervention, formulation of intervention, control, dosage, and medication period; and (4) outcomes: outcomes, main conclusion, and adverse events.

### 2.4. Assessment with Risk of Bias

Two authors (S. Jang and Y. Ko) assessed methodological quality using the Risk of Bias (RoB) tool, which was developed by Cochrane [7]. RoB was divided into 6 selection biases, including 2 selection biases (random sequence generation and allocation concealment), performance bias, detection bias, attrition bias, and reporting bias. Each item of all the included RCTs was determined as “high risk,” “unclear,” or “low risk.” A RoB graph was drawn using RevMan 5.3 program.

### 2.5. Data Analysis

We used mean difference (MD) with 95% confidential interval (CI) to measure primary outcomes between trials. Analyses were divided into 4 subgroups depending on type of controls: no treatment, placebo, metformin, other western medicines. Heterogeneity was analyzed by the Cochrane *Q* and *I* ^2^ test. *I* ^2^ values of 25%, 50%, and 75% mean low, medium, and high levels of statistical heterogeneity. RevMan 5.3 program was used for analysis. We also made summary of findings (SoF) table to present results of review by Gradepro software.

## 3. Results

### 3.1. Description of Included Trials

From ten databases, 1,098 literature articles were identified. Among them, 826 records remained after eliminating duplications, and 733 records were excluded after screening titles and abstracts. By reviewing full texts of 93 records, 12 RCTs were included in this systematic review. The process of the study selection is shown in Figure 1.

#### 3.1.1. Characteristics of Study

Among the 12 included RCTs [13–24], 11 studies [14–24] were written in Chinese and conducted in China, and 1 study [13] was written in English and conducted in India. Four trials [15, 16, 19, 24] selected the IDF guideline for diagnosis of metabolic syndrome, 2 trials [13, 22] used the NCEP-ATP guideline, 3 trials [17, 20, 23] used the Chinese Diabetes Society criteria, and 3 trials used the American Diabetes Association guideline [18], the AACE clinical criteria [21], and the China Dyslipidemia Prevention guideline [14], respectively. Six trials [15, 17–20, 24] additionally applied Chinese pattern identification as the inclusion criterion. There were 4 trials [15, 17, 19, 20] that had a medication period of less than 8 weeks, 3 trials [18, 21, 23] with a medication period of more than 8 weeks but less than 12 weeks, and 5 trials [13, 14, 16, 22, 24] with a medication period of more than 12 weeks. Details are described in Table 2.

#### 3.1.2. Participants

The number of participants for the trials varied from 43 [14] to 183 [13]. No trial was restricted to participants based on sex, but one trial [17] did not report sex distribution. Six trials included Chinese pattern identification as inclusion criteria: 2 trials [15, 19] of Exuberance of Phlegm-Dampness Type, 1 trial [24] of Spleen Deficiency and Stagnation of Dampness Type, 1 trial [18] of Heart-Liver Stagnated Heat Type, 1 trial [20] of Flaming-Up of Fire of the Liver Type, and 1 trial [17] of Blood-Stasis Type. Two trials [14, 20] were conducted on patients with hypertension and metabolic syndrome.

#### 3.1.3. Comparisons

Comparisons were divided into two types. One type was a placebo or no treatment, and 2 trials [13, 14] were compared with a placebo, while 3 trials [15–17] were conducted under no treatment. Diet-control and exercise or health education was used for the no treatment group. The other type was western medicine, and 7 trials [18–24] followed this comparison type. Metformin was used as a comparison in 3 trials [18, 19, 21], and nifedipine was used in 1 trial [20]. Two trials [22–24] provided different conventional medicines according to the symptoms of the patients.

### 3.2. Effects of Interventions

**Figure 2:**
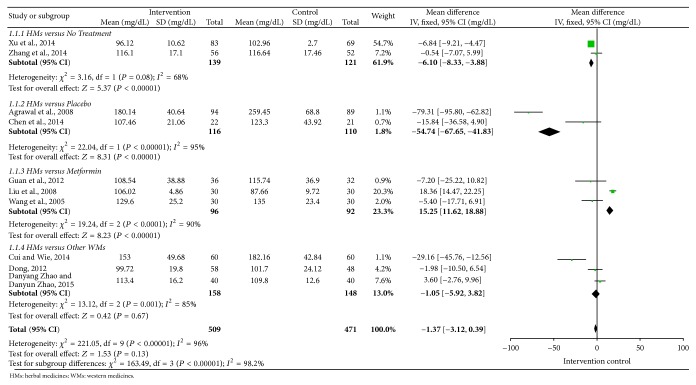
Forest plot for fasting plasma glucose (FPG).

**Figure 3 fig3:**
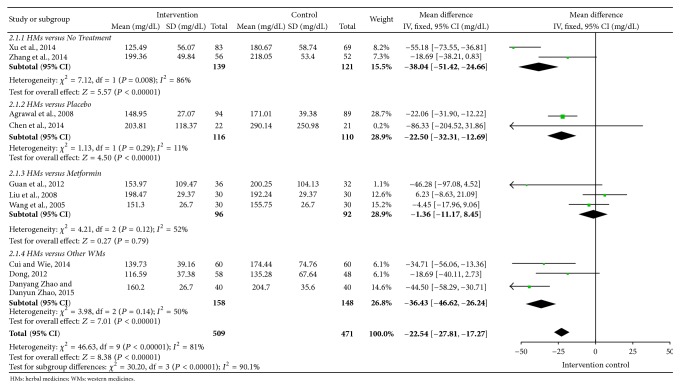
Forest plot for triglycerides (TG).

**Figure 4 fig4:**
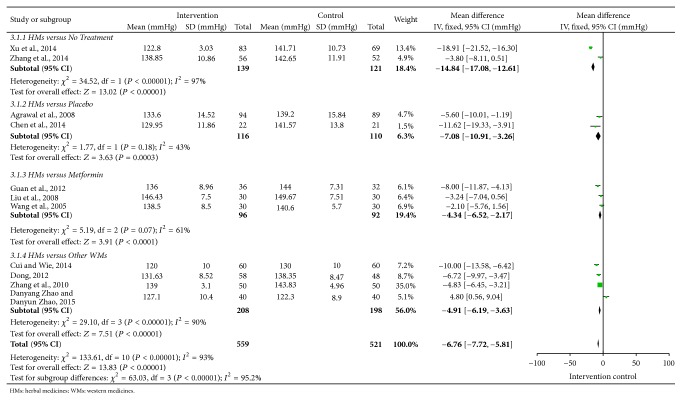
Forest plot for systolic blood pressure (SBP).

**Figure 5:**
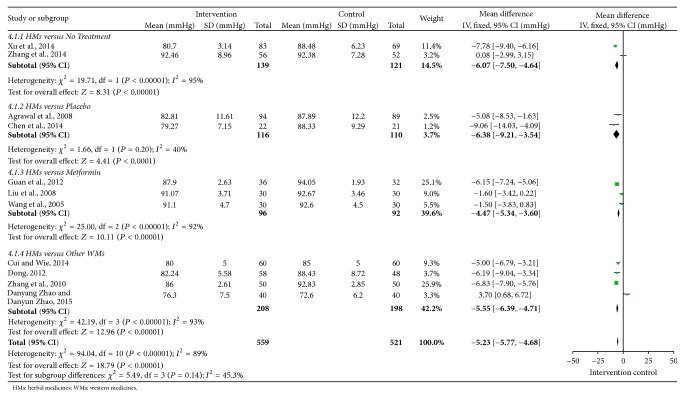
Forest plot for diastolic blood pressure (DBP).

Overall efficacy of FPG, TG, SBP, DBP, WC, and HDL-C was presented in summary of findings (Table 3). Meta-analyses of FPG, TG, SBP, and DBP were shown in Figures 2 [Fig fig3] [Fig fig4]–5. All 12 trials had different herbal medicine interventions; therefore, we also compared effects of each intervention.  Table 4 shows the mean differences (MD) for each outcome measure. The unit mmol/L was converted into mg/dL.

#### 3.2.1. Waist Circumference and Body Mass Index

There were seven trials [13–16, 19, 20, 24] reporting WC. Five trials [14–16, 20, 24] showed significant reductions of WC compared with the control groups: Yiqi Huaju Recipe (MD: −4.68, *n* = 43), Daotan decoction (MD: −2.98, *n* = 108), Gegen Shanzha decoction (MD: −5.31, *n* = 152), Pinggan Jianya pill (MD: −7.81, *n* = 100), and Shenling Jianpihuashi decoction (MD: −7.2, *n* = 80). Modified Banxia Baizhu Tianma decoction [19] showed less effect on decreasing WC than metformin. There was a slight increase in one trial [13]; however, it was not statistically significant.

There were nine trials [13–16, 19–21, 23, 24] reporting BMI. Six trials [13–16, 20, 23] showed significant reductions of BMI compared with the control groups: Dia-No decoction (MD: −0.18, *n* = 183), Yiqi Huaju Recipe (MD: −1.51, *n* = 43), Daotan decoction (MD: −0.49, *n* = 108), Gegen Shanzha decoction (MD: −2.69, *n* = 152), Pinggan Jiangya pill (MD: −0.74, *n* = 100), and Shengjiangtongmai powder (MD: −2.52, *n* = 120). There were significant reductions after treatment in the remaining 3 trials [19, 21, 24]; however, it was not significant when compared with the control groups. These trials included the Modified Banxia Baizhu Tianma decoction (MD: −0.74, *n* = 60), Huanglian Wendan decoction (MD: −1.95, *n* = 68), and Shenling Jianpihuashi decoction (MD: −2.7, *n* = 80).

#### 3.2.2. Blood Glucose

There were 10 trials [13–16, 18, 19, 21–24] reporting FPG and 4 trials [14, 18, 19, 24] reporting 2 hPG. Three trials [13, 16, 23] showed significant reductions of FPG compared with the control groups: Dia-No decoction (MD: −41.15, *n* = 183), Gegen Shanzha decoction (MD: −21.42, *n* = 152), and Shengjiangtongmai powder (MD: −82.08, *n* = 120). Five trials had an effect on lowering FPG; however, effects of herbal medicines were not more than the controls: Yiqi Huaju Recipe (MD: −7.02, *n* = 43), Qinggan Jiangtang tablet (MD: −28.8, *n* = 60), Modified Banxia Baizhu Tianma decoction (MD: −7.02, *n* = 60), Huanglian Wendan decoction (MD: −27.54, *n* = 68), and Shenling Jianpihuashi decoction (MD: −16.2, *n* = 80). Daotan decoction [15] and Xueguan Ruanhua decoction [22] were not effective in decreasing FPG. As shown in Figure 2, mean FPG in the intervention groups was 1.37 lower than control groups within 10 trials (−3.13 to 0.39).

For 2 hPG, four trials [14, 18, 19, 24] showed significant reductions after treatment, and 1 trial [14] demonstrated an effect when compared with the control group: Yiqi Huaju Recipe (MD: −29.34, *n* = 43).

#### 3.2.3. Blood Lipids

There were 10 trials [13–16, 18, 19, 21–24] reporting TG and 9 trials [13–16, 18, 19, 21, 22, 24] reporting HDL-C. Nine trials [13, 15, 16, 18, 19, 21–24] showed significant reductions of TG compared with the control groups: Dia-No decoction (MD: −10.59, *n* = 183), Daotan decoction (MD: −28.48, *n* = 108), Gegen Shanzha decoction (MD: −99.68, *n* = 152), Qinggan Jiangtang tablet (MD: −12.46, *n* = 60), Modified Banxia Baizhu Tianma decoction (MD: −23.14, *n* = 60), Huanglian Wendan decoction (MD: −62.3, *n* = 68), Xueguan Ruanhua decoction (MD: −30.26, *n* = 106), Shengjiangtongmai powder (MD: −71.2, *n* = 120), and Shenling Jianpihuashi decoction (MD: −89.89, *n* = 80). As shown in Figure 3, mean TG in the intervention groups was 22.54 lower than control groups within 10 trials (−27.81 to −17.27).

Five trials [15, 16, 19, 21, 22] demonstrated significant increases of HDL-C: Daotan decoction (MD: +2.32, *n* = 108), Gegen Shanzha decoction (MD: +18.91, *n* = 152), Modified Banxia Baizhu Tianma decoction (MD: +7.72, *n* = 60), Huanglian Wendan decoction (MD: +11.19, *n* = 68), and Xueguan Ruanhua decoction (MD: +15.83, *n* = 106). Yiqi Huaju Recipe [14] was not effective in improving either TG or HDL-C.

#### 3.2.4. Blood Pressure

There were 11 trials [13–16, 18–24] reporting systolic and diastolic blood pressure. All 11 trials showed significant decreases in blood pressure: Dia-No decoction (MD: −2.29/−1.23, *n* = 183), Yiqi Huaju Recipe (MD: −11.32/−6.5, *n* = 43), Daotan decoction (MD: −7.43/−2.28, *n* = 108), Gegen Shanzha decoction (MD: −19.66/−10.5, *n* = 152), Qinggan Jiangtang tablet (MD: −7.5/−2.9, *n* = 60), Modified Banxia Baizhu Tianma decoction (MD: −4.38/−3.23, *n* = 60), Pinggan Jiangya pill (MD: −28.00/−14.33, *n* = 100), Huanglian Wendan decoction (MD: −10.73/−8.24, *n* = 68), Xueguan Ruanhua decoction (MD: −8.75/−9.33, *n* = 106), Shengjiangtongmai powder (MD: −30/−15, *n* = 120), and Shenling Jianpihuashi decoction (MD: −11.6/−9.3, *n* = 80). Ten herbal medicines (except Shenling Jianpihuashi decoction [24]) had more effect than the controls, and Shenling Jianpihuashi decoction was not inferior to nifedipine.

The mean SBP was 6.76 lower in the intervention groups compared to control groups within 11 trials (−7.72 to −5.81) (Figure 4). The mean DBP was 5.23 lower in the intervention groups than control groups within 11 trials (−5.77 to −4.86) (Figure 5).

### 3.3. Adverse Events and Safety

Six RCTs [13, 14, 20–22, 24] reported 26 adverse events. Nine cases occurred in the herbal medicine group, and the remaining 17 cases occurred in the western medicine group. There was no adverse event in the placebo or no treatment control group. The Dia-No group [13] had 6 upper digestive disorders, the Huanglian Wendan decoction group [21] had 1 gastrointestinal disorder, and the Yiqi Huaju Recipe group [14] had 2 skin hypersensitivities. There was no observed adverse event in the Pinggan Jiangya pill group [20], the Xueguan Ruanhua decoction group [22], and the Shenling Jianpihuashi decoction group [24]. The most commonly reported symptoms were digestive disorders such as nausea, vomiting, and burning of the epigastrium (Table 5).

### 3.4. Assessment with Risk of Bias

**Figure 6:**
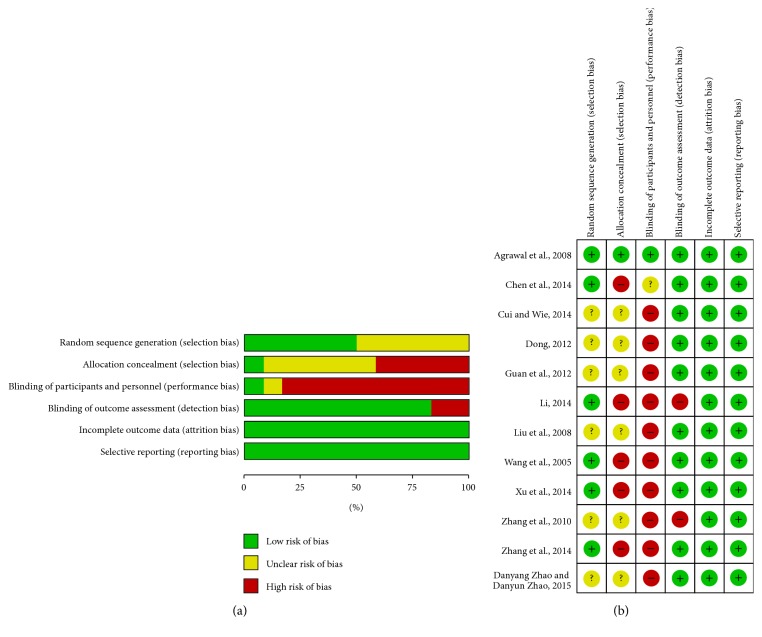
(a) Risk of bias graph: reviewers' assessments about each risk of bias item presented as percentages of all included studies. (b) Risk of bias summary: assessments about each risk of bias item for each included study. “+”: low risk, “?”: unclear risk, and “−”: high risk.

RoB of the 12 RCTs was assessed into 6 areas. Six RCTs [13–18] used a random number table to generate the random sequence. There were high risks of performance bias (blinding of participants and personnel) in 10 trials [15–24] due to the difference of drug formulation. Except in 2 RCTs [17, 20] where the primary outcomes were blood pressure and blood-stasis symptom, the remaining 10 trials were assessed as “low risk” for detection bias (blinding of outcome assessment). There were low risks of attrition bias (incomplete outcome data) and reporting bias (selective reporting) in all 12 trials. Only 1 RCT [13] was assessed as “low risk” for all six items. Details of RoB are presented in Figure 6.

## 4. Discussion

As a result of searching 10 databases, 12 randomized controlled trials were included in the systematic review. Because the review's purpose was to determine the efficacy and safety of herbal medicines for metabolic syndrome, clinical trials that included herbal medicines combined with conventional western medicines were excluded. Five trials [13–17] studied herbal medicines versus a placebo or no treatment, and seven trials [18–24] studied herbal medicines versus western medicines. All 12 trials included controls for diet-control, exercise, or health education with medications.

According to Table 4, all the trials showed positive effects with the administration of herbal medicines, and most of them proved significant. Gegen Shanzha decoction [16] improved 5 metabolic indexes, including WC, FPG, TG, HDL-C, and BP. Yiqi Huaju Recipe [14] had an effect on lowering body weight, blood sugar, and blood pressure (except blood lipids). Because the outcome measured the blood-stasis symptom only in the trial for Xuefu Zhuyu decoction [17], the efficacy of Xuefu Zhuyu decoction on metabolic diseases could not be determined. The Pinggan Jiangya pill [20] was effective for the metabolic syndrome of Flaming-Up of Fire of the Liver Type. Flaming-Up of Fire of the Liver Type is the largest type of hypertension [25]; therefore, the Pinggan Jiangya pill would be suitable to treat obesity and hypertension. The Qinggan Jiangtang tablet [18], Modified Banxia Baizhu Tianma decoction [19], Huanglian Wendan decoction [21], Xueguan Ruanhua decoction [22], the Shengjiangtongmai powder [23], and Shenling Jianpihuashi decoction [24] were superior (or not inferior) to western medicines used to treat metabolic syndrome.

As for summary of findings (Table 3), mean differences of metabolic parameters were compared. Metabolic syndrome is not determined by single indicator, and comparing value of each parameter is not appropriate, strictly speaking. Relative risk (RR) of metabolic syndrome should be calculated. However, there was no study presenting difference of prevalence before and after treatment. Meanwhile, WC and HDL-C could not be calculated because they were not separated by men and women within included trials.

Forest plots of FPG, TG, SBP, and DBP indicate high heterogeneity although subgroup analysis was done (Figures 2 [Fig fig3] [Fig fig4]–5). It is assumed that heterogeneity did not result from controls. Instead, difference of each intervention would have been affected. It is also limitation of meta-analysis in this review.

Regarding safety, adverse events were reported less in herbal medicines than in western medicines (Table 5). In Zhang et al.'s trial [20], the nifedipine group had 4 facial flushes, while the Pinggan Jiangya group had no adverse events. Additionally, there was 1 gastrointestinal disorder with Huanglian Wendan decoction, but there were 5 adverse events when metformin was used in Guan et al.'s trial [21]. Therefore, herbal medicines would be an effective and safe treatment for metabolic syndrome compared with western medicines.

In 6 trials [15, 17–20, 24], oriental pattern identification along with metabolic factors was set for inclusion criteria. A selection of treatments with pattern identifications would help reduce metabolic risk factors, improve general conditions, and decrease chances of adverse events. With the collection of such trial data, this would provide a ground for herbal medicines to be used as treatment for obesity or metabolic syndrome by pattern identifications.

There are more studies on herbal medicines for metabolic syndrome although they were not included in this review. Keishibukuryogan [26], Yiqi Huaju Qingli Formula [27], Ba-Wei-Wan [28], Heqi San [29], Baoling decoction [30], and Combination of Four Gentlemen Decoction and Sini Powder [31] showed effects on metabolic syndrome; however, trials on these were excluded because treatment group was also treated with conventional western medicines. Herbal supplements, for example, Ginseng [32], berberine, bitter melon [33], nigella sativa [34], and* Gymnema sylvestre* [35], are also used for management of metabolic diseases. In particular it is well known that ginsenosides which are compounds of ginseng have clear effect of regulating blood glucose and blood pressure [33].

This review has some limitations. First, metabolic syndrome is not a single disorder but rather a complex disease. The herbal medicines used as interventions and their efficacy need to be matched for the following indicators: waist circumference, body weight, blood glucose, blood lipids, and blood pressure. Second, the trials included in the study showed a relatively low level of quality because most of them failed to conduct a double-blinded technique, and only 1 trial [13] met the qualification for advanced protocol. Because of these limitations, there may be the possibility that therapeutic effects have been overestimated. Publication bias also needs to be taken into consideration. Third, there could be trials missing even though we tried to cover all of the RCTs from English, Korean, Chinese, and Japanese databases. However, the study is significant because it has reviewed RCTs on the administration of herbal medicines for treating metabolic diseases. Further studies are needed to develop new herbal medicines for metabolic syndrome and to build evidence on their effectiveness and safety.

## 5. Conclusion

Herbal medicines showed therapeutic effects on regulating waist circumference, blood glucose, blood lipids, and blood pressure in this systematic review. This means herbal medicines have the potential to be complementary and alternative medicines for metabolic syndrome. However, more high quality trials are needed to prove the efficacy and safety of herbal medicines.

## Figures and Tables

**Table 1:** Search strategy (PubMed).

Chinese herbal medicine	
#1	Chinese medic^*∗*^
#2	TCMs or TCM
#3	Chinese herb^*∗*^
#4	Chinese drug^*∗*^
#5	Chinese formul^*∗*^
#6	Chinese plant^*∗*^
#7	Chinese prescri^*∗*^
#8	Chinese remed^*∗*^
#9	Chinese materia^*∗*^ medica^*∗*^
#10	kampo
#11	herb^*∗*^ medic^*∗*^
#12	“Medicine, Chinese traditional” (MeSH Terms)
#13	“Medicine, East Asian Traditional” (MeSH Terms)
#14	“Medicine, kampo” (MeSH Terms)
#15	“Herbal Medicine” (MeSH Terms)
#16	“drugs, Chinese herbal” (MeSH Terms)
#17	#1 or #2 or #3 or #4 or #5 or #6 or #7 or #8 or #9 or #10 or #11 or #12 or #13 or #14 or #15 or #16

Metabolic syndrome	

#18	“Metabolic Syndrome X” (MeSH Terms)
#19	metabolic syndrome
#20	cardiometabolic syndrome
#21	insulin resistance syndrome
#22	“syndrome X”
#23	“Reaven's syndrome”
#24	CHAOS AND Australia
#25	#18 or #19 or #20 or #21 or #22 or #23 or #24

Randomized controlled trials	

#26	((clinical (Title/Abstract) AND trial (Title/Abstract)) OR clinical trials (MeSH Terms) OR clinical trial (Publication Type) OR random^*∗*^ (Title/Abstract) OR random allocation (MeSH Terms) OR therapeutic use (MeSH Subheadings))
#27	#17 AND #25 AND #26

**Table 2:** Characteristics of RCTs using herbal medicine in the treatment of metabolic syndrome.

Author, year, country	Sample size (I/C)	Age	Sex (M/F)	Inclusion criteria of MetS	Chinese pattern identification	Intervention	Control	Period	Outcomes	Main conclusion
Agrawal et al., 2008, India [13]	94/89	I: 51.85 ± 11.8 C: 52.81 ± 10.3	103/80	NCEP-ATP guideline (2002)	ND	Dia-No, 1 tab, bid	Placebo, 1 tab, bid	3 m	WC, FPG, HbA1c, TC, TG, HDL-C, VLDL, LDL-C, SBP, DBP, urea, creatinine, bilirubin, GOT, GPT	Dia-No is safe and effective drug in the management of MetS.

Chen et al., 2014, China [14]	22/21	I: 48.73 ± 9.4 C: 48.90 ± 8.9	28/15	China Dyslipidemia Prevention guideline (2007)	ND	Yiqi Huaju Formula	Placebo, bid	12 w	BMI, body weight, WC, HC, WHR, FPG, HbA1c, FPI, 2 hPG, HOMA-IR, TC, TG, HDL-C, LDL-C, 24 hBP, dBP, nBP, 24 hBPv, dBPv, nBPv, 24 hBPp, dBPp, nBPp	Yiqi Huaju Formula combining with diet-control and exercise has effect on reducing blood pressure.

Zhang et al., 2014, China [15]	56/52	61.5 (37~80)	59/49	IDF guideline (2005)	Phlegm-Dampness Type	Daotan decoction	No treatment	4 w	BMI, WC, TG, HDL-C, FPG, SBP, DBP	Daotan decoction can ameliorate the blood lipid and blood pressure of MetS of phlegm-dampness constitution.

Xu et al., 2014, China [16]	83/69	I: 51.75 ± 10.1 C: 52.30 ± 14.3	70/82	IDF guideline (2005)	ND	Gegen shanzha decoction	No treatment	6 m	BMI, body weight, WC, AC, HC, WHR, SFA, VFA, FPG, FINS, HOMA-IR, TC, TG, HDL-C, LDL-C, SBP, DBP, SF-36	Gegen Shanzha decoction combining with TCM physique recuperation is effective for MetS.

Li, 2014, China [17]	32/30	NR	NR	Chinese Diabetes Society criteria (2004)	Blood-Stasis Type	Xuefu zhuyu decoction, bid	No treatment	4 w	Blood stasis symptoms	Xuefu Zhuyu decoction has clinical efficacy for MetS of Blood-Stasis Type.

Wang et al., 2005, China [18]	30/30	I: 49.6 ± 7.9 C: 50.6 ± 7.6	27/33	American Diabetes Association guideline (1997)	Heart-Liver Stagnated Heat Type	Qinggan Jiangtang, 3 tabs, bid	Glucophage (metformin), 3 tabs, bid	2 m	FPG, 2 hPG, HbA1c, TC, TG, HDL-C, LDL-C, SBP, DBP, FINS, PINS, F-CP, P-CP, HOMA-IR, HOMA-*β*, FFA,	Qinggan Jiangtang tablet has effects on reducing blood glucose, blood lipids, blood pressure, and insulin resistance.

Liu et al., 2008, China [19]	30/30	30–60	NR	IDF guideline (2005)	Exuberance of Phlegm-Dampness Type	Modified Banxia Baizhu Tianma decoction, 120 mL, bid	Metformin, 0.25 mg, tid	6 w	WC, BMI, FPG, 2 hPG, FINS, ISI, TC, TG, HDL-C, LDL-C, ApoA1, ApoB, SBP, DBP, effective rate, Chinese symptoms	Modified Banxia Baizhu Tianma Decoction has effects on treating MetS of Exuberance of Phlegm-Dampness Type.

Zhang et al., 2010, China [20]	50/50	I: 52.61 ± 5.4 C: 51.10 ± 12.2	80/20	Chinese Diabetes Society criteria (2004)	Flaming-Up of Fire of the Liver Type	Pinggan Jiangya pill, 6 g, tid	Nifedipine, 10 mg, tid	1 m	BMI, body weight, WC, SBP, DBP	Pinggan Jiangya pill has effects on decreasing blood pressure and weight of MetS of Flaming-Up of Fire of the Liver Type.

Guan et al., 2012, China [21]	36/32	47.2 ± 15.4	39/29	AACE clinical criteria (2003)	ND	Huanglian Wendan decoction	Metformin, 0.25 mg, bid	8 w	BMI, FPG, TC, TG, HDL-C, LDL-C, SBP, DBP	Huanglian Wendan decoction is the same as metformin in reducing weight and blood sugar but better in decreasing blood lipids and blood pressure.

Dong, 2012, China [22]	58/48	I: 24~78 C: 21~73	44/62	NCEP ATP guideline (2002)	ND	Xueguan Ruanhua decoction (1 m) → Xueguan Ruanhua pill (2 m)	① DM: metformin or rosiglitazone ② HL: simvastatin or fenofibric acid ③ HTN: captopril or nifedipine	3 m	FPG, TG, HDL-C, SBP, DBP, effective rate	Xueguan Ruanhua decoction has effects on the treatment of MetS.

Cui and Wie, 2014, China [23]	60/60	I: 51.3 ± 6.0 C: 52.3 ± 5.4	69/51	Chinese Diabetes Society criteria (2004)	ND	Shengjiangtongmai powder, 300 mL, bid	① Metformin, 0.25 mg, bid ② Simvastatin, 10 mg, qd ③ Enalapril, 10 mg, qd	2 m	BMI, SBP, DBP, TC, TG, FPG, effective rate	Shengjiangtongmai powder can effectively improve blood glucose, blood lipid, blood pressure, and obesity.

Danyang Zhao and Danun Zhao, 2015, China [24]	40/40	I: 48.7 ± 12.5 C: 47.6 ± 12.6	NR	IDF guideline (2005)	Spleen Deficiency and Stagnation of Dampness Type	Shenling Jianpihuashi decoction, bid	① DM: metformin, 500 mg, tid ② HTN: losartan, 50 mg, qd	12 w	WC, BMI, FPG, 2 hPG, HbA1c, TC, TG, HDL-C, LDL-C, SBP, DBP, HOMA-IR, effective rate	Shenling Jianpihuashi decoction can effectively improve MetS of Spleen Deficiency and Stagnation of Dampness type.

I: intervention group; C: control group; M: male; F: female; NR: not reported; MetS: metabolic syndrome; DM: diabetes mellitus; HTN: hypertension; HL: hyperlipidemia; WC: waist circumference; FPG: fasting plasma glucose; TC: total cholesterol; HDL-C: high density lipoprotein cholesterol; VLDL: very low density lipoprotein; LDL-C: low density lipoprotein cholesterol; SBP: systolic blood pressure; DBP: diastolic blood pressure; GOT: glutamic oxaloacetic transaminase; GPT: glutamic-pyruvic transaminase; BMI: body mass index; HC: hip circumference; WHR: waist hip ratio; FPI: fasting plasma insulin; 2hPG: 2-hour postprandial glucose; TG: triglycerides; AC: arm circumference; SFA: subcutaneous fat area; VFA: visceral fat area; FINS: fasting serum insulin; PINS: postprandial serum insulin; F-CP: fasting serum C-peptide; P-CP: postprandial serum C-peptide; FFA: free fatty acids; ISI: insulin sensitivity index.

**Table 3:** Summary of findings in this systematic review.

Herbal medicines compared to controls for metabolic syndrome						
*Patient or population*: metabolic syndrome						
*Setting*: outpatient and inpatient						
*Intervention*: herbal medicines						
*Comparison*: no treatment, placebo, and western medicines						

Outcomes	Anticipated absolute effects^*∗*^ (95% CI)		Relative effect (95% CI)	Number of participants (studies)	Quality of the evidence (grade)	Comments
	Risk with control	Risk with intervention				

Fasting plasma glucose (FPG)	The mean FPG ranged across control groups from 87 to 260 mg/dL	The mean FPG in the intervention groups was 1.37 mg/dL lower (3.12 lower to 0.39 higher)	—	980 (10 RCTs)	⨁⨁◯◯ Low^1^	Lower score indicates less risk of diabetes mellitus.
Triglycerides (TG)	The mean TG ranged across control groups from 135 to 291 mg/dL	The mean TG in the intervention groups was 22.54 mg/dL lower (27.81 lower to 17.27 lower)	—	980 (10 RCTs)	⨁⨁⨁◯ Moderate^2^	Lower score indicates less risk of dyslipidemia.
Systolic blood pressure (SBP)	The mean SBP ranged across control groups from 122 to 150 mmHg	The mean SBP in the intervention groups was 6.76 mmHg lower (7.72 lower to 5.81 lower)	—	1080 (11 RCTs)	⨁⨁⨁◯ Moderate^3^	Lower score indicates less risk of hypertension.
Diastolic blood pressure (DBP)	The mean DBP ranged across control groups from 72 to 95 mmHg	The mean DBP in the intervention groups was 5.23 mmHg lower (4.77 lower to 4.68 lower)	—	1080 (11 RCTs)	⨁⨁⨁◯ Moderate^3^	Lower score indicates less risk of hypertension.
Waist circumference (WC)	See comment		—	726 (7 RCTs)	⨁⨁◯◯ Low	Only 2 studies showed WC separated by sex, so risk could not be calculated.
High density lipoprotein cholesterol (HDL-C)	See comment		—	860 (9 RCTs)	⨁⨁◯◯ Low	No study showed HDL-C separated by sex, so risk could not be calculated.

^*∗*^The risk in the intervention group (and its 95% confidence interval) is based on the assumed risk in the comparison group and the relative effect of the intervention (and its 95% CI).

CI: confidence interval; MD: mean difference.

^1^Heterogeneity and possible publication bias downgraded quality of the evidence.

^2^Sparse data downgraded quality of the evidence.

^3^Heterogeneity downgraded quality of the evidence.

**Table 4:** Estimate effects of herbal medicines for metabolic syndrome: differences of values before and after treatment.

Intervention	Study ID	WC	BMI	FPG	2 hPG	TG	HDL-C	SBP	DBP
Dia-No	Agrawal et al., 2008 [13]	+0.02	−0.18^*∗*^	−41.15^*∗*^	NR	−10.59^*∗*^	+1.12	−2.29^*∗*^	−1.23^*∗*^
Yiqi Huaju Recipe	Chen et al., 2014 [14]	−4.68^*∗*^	−1.51^*∗*^	−7.02^#^	−29.34^*∗*^	−7.12	+1.93	−11.32^*∗*^	−6.5^*∗*^
Daotan decoction	Zhang et al., 2014 [15]	−2.98^*∗*^	−0.49^*∗*^	−2.52	NR	−28.48^*∗*^	+2.32^*∗*^	−7.43^*∗*^	−2.28^*∗*^
Gegen Shanzha decoction	Xu et al., 2014 [16]	−5.31^*∗*^	−2.69^*∗*^	−21.42^*∗*^	NR	−99.68^*∗*^	+18.91^*∗*^	−19.66^*∗*^	−10.5^*∗*^
Xuefu Zhuyu decoction	Li, 2014 [17]	NR	NR	NR	NR	NR	NR	NR	NR
Qinggan Jiangtang tablet	Wang et al., 2005 [18]	NR	NR	−28.8^#^	−52.2^#^	−12.46^*∗*^	+2.70	−7.5^*∗*^	−2.9^*∗*^
Modified Banxia Baizhu Tianma decoction	Liu et al., 2008 [19]	−0.91	−0.74^#^	−7.02^^#^^	−19.08^#^	−23.14^*∗*^	+7.72^*∗*^	−4.38^*∗*^	−3.23^*∗*^
Pinggan Jiangya pill	Zhang et al., 2010 [20]	−7.81^*∗*^	−2.95^*∗*^	NR	NR	NR	NR	−28.00^*∗*^	−14.33^*∗*^
Huanglian Wendan decoction	Guan et al., 2012 [21]	NR	−1.95^#^	−27.54^#^	NR	−62.3^*∗*^	+11.19^*∗*^	−10.73^*∗*^	−8.24^*∗*^
Xueguan Ruanhua decoction	Dong, 2012 [22]	NR	NR	−6.12	NR	−30.26^*∗*^	+15.83^*∗*^	−8.75^*∗*^	−9.33^*∗*^
Shengjiangtongmai powder	Cui and Wie, 2014 [23]	NR	−2.52	−82.08^*∗*^	NR	−89.89^*∗*^	NR	30^*∗*^	15^*∗*^
Shenling Jianpihuashi decoction	Danyang Zhao and Danun Zhao, 2015 [24]	−7.2^*∗*^	−2.7^#^	−16.2^#^	−34.2^#^	−71.2^*∗*^	+3.86	−11.6^#^	−9.3^#^

^*∗*^Significant difference between intervention and control group (*P* < 0.05).

^#^Significant difference before and after treatment (*P* < 0.05).

WC: waist circumference; BMI: body mass index; FPG: fasting plasma glucose; 2 hPG: 2-hour postprandial glucose; TG: triglycerides; HDL-C: high density lipoprotein cholesterol; SBP: systolic blood pressure; DBP: diastolic blood pressure.

**Table 5:** Compositions of herbal medicines and adverse events in the included RCTs.

Intervention	Study ID	Compositions	Formulation	Adverse events
Dia-No	Agrawal et al., 2008 [13]	*Syzygium cumini *20%,* Gymnema sylvestre *20%, *Trigonella foenumgraecum 14%, Emblica officinalis 10%, Azadirachta indica 7%, Cassia auriculata 7%, Tribulus terrestris 7%, Andrographis paniculata 5%, Pterocarpus marsupium 5%*, and* Momordica charantia *5%	Tablet	Nausea 2, vomiting 1, loss of appetite 2, and burning epigastrium 1 in intervention group

Yiqi Huaju Recipe	Chen et al., 2014 [14]	*Astragalus membranaceus, Coptis chinensis, Typha orientalis, Alisma canaliculatum, Artemisia capillaris, *and so forth	Decoction	Skin hypersensitivity 2 in intervention group

Daotan decoction	Zhang et al., 2014 [15]	*Pinellia ternata *10 g*, Arisaema erubescens *Schott 5 g*, Citrus reticulata *10 g*, Citrus sinensis *10 g*, Poria cocos* 15 g, *Zingiber officinale *5 g, and* Glycyrrhiza uralensis* 5 g	Decoction	Not reported

Gegen Shanzha decoction	Xu et al., 2014 [16]	*Pueraria montana* and *Crataegus pinnatifida *Bunge each 10~20 g (1 : 1)	Decoction	Not reported

Xuefu Zhuyu decoction	Li, 2014 [17]	*Prunus persica *(L.) Batsch 9 g* Carthamus tinctorius* 9 g,* Rehmannia glutinosa* 9 g, *Ligusticum officinale* Kitag. 5 g, *Angelica sinensis *9 g, *Paeonia lactiflora *Pallas 6 g, *Achyranthes japonica* Nakai 9 g, *Platycodon grandiflorum *5 g, *Bupleurum falcatum* 3 g, *Citrus aurantium* L. 6 g, and *Glycyrrhiza uralensis* 3 g	Decoction	Not reported

Qinggan Jiangtang tablet	Wang et al., 2005 [18]	*Bupleurum falcatum* 8 g, *Gardenia jasminoides* 10 g, *Coptis chinensis* 4 g, *Scutellaria baicalensis* 8 g, *Rehmannia glutinosa* 15 g, *Lilium longiflorum* 20 g, *Anemarrhena asphodeloides* Bunge 10 g, pollen 20 g, *Gastrodia elata* Blume 10 g, and *Cassia occidentalis* L. 20 g	Tablet	Not reported

Modified Banxia Baizhu Tianma decoction	Liu et al., 2008 [19]	*Pinellia ternata* 9 g, *Gastrodia elata* Blume 6 g, *Pueraria montana* 20 g, *Atractylodes macrocephala* Koidzumi 15 g, *Alisma canaliculatum* 30 g, *Pleuropterus multlforus* 15 g, *Crataegus pinnatifida* Bunge 15 g, *Salviae miltiorrhizae* Radix 25 g, *Astragalus membranaceus* 30 g, *Poria cocos* 15 g, *Cassia obtusifolia *L. 15 g, and *Citrus reticulata* 10 g,	Decoction	Not reported

Pinggan Jiangya pill	Zhang et al., 2010 [20]	*Prunella vulgaris* 24 g*, Uncaria rhynchophylla *20 g*, Saiga tataria *L. 2 g, *Folium ilicis* (Kuding tea) 10 g, *Sophora japonica* L. 10 g, *Pteria martensii *30 g, *Tribulus terrestris *L. 20 g, magnetitum 20 g, *Scutellaria baicalensis* 15 g, *Cassia obtusifolia* L. 20 g, *Achyranthes japonica *Nakai 15 g	Pill	Facial flush 4 in control group

Huanglian Wendan decoction	Guan et al., 2012 [21]	*Coptis chinensis*, *Pinellia ternata*, *Citrus reticulata, Poria cocos, Pueraria montana, Cassia obtusifolia* L., *Astragalus membranaceus, Phyllostachys bambusoides *Sieb. et Zucc.,* Salviae miltiorrhizae *Radix, and* Glycyrrhiza uralensis*	Decoction	Gastrointestinal disorder 1 in intervention group, abdominal pain and vomiting 4, and weakness 1 in control group

Xueguan Ruanhua decoction	Dong, 2012 [22]	*Taxillus chinensis, Apocynum cannabinum, Angelica sinensis, Paeonia lactiflora* Pallas, *Ligusticum officinale* Kitag., *Gastrodia elata *Blume, *Eucommia ulmoides *Oliver, *Salviae miltiorrhizae *Radix, *Vitex rotundifolia* L., *Chrysanthemum morifolium, Hirudo nipponica *Whitman, mulberry leaf, *Coptis chinensis*, *Pueraria montana, Fritillaria cirrhosa* D. Don, and so forth	Decoction	Nausea and vomiting 2, hypoglycemia 1, and hypotension 3 in control group

Shengjiangtongmai powder	Cui and Wie, 2014 [23]	*Bombyx mori *10 g*, Cryptotympana coreana *10 g*, Curcuma longa *9 g, *Rheum palmatum *12 g,* Coptis chinensis *6 g, *Panax quinquefolius *25 g*, Atractylodes japonica *15 g, *Pinellia ternata *12 g,* Trichosanthes kirilowii *Maxim.15 g	Decoction	Not reported

Shenling Jianpihuashi decoction	Danyang Zhao and Danun Zhao, 2015 [24]	*Codonopsis tangshen* Oliver 15 g, *Astragalus membranaceus* 15 g, *Poria cocos* 15 g, *Atractylodes macrocephala *Koidzumi 15 g, *Dioscorea opposita* 15 g, *Coixlachrymajobi *var. mayuen 15 g, *Amomum xanthioides* 6 g, *Nelumbo nucifera *10 g, *Alisma canaliculatum* 15 g, and *Panax notoginseng* 3 g	Decoction	Gastrointestinal disorder 2 in control group
